# Susceptibility of Well-Differentiated Airway Epithelial Cell Cultures from Domestic and Wild Animals to Severe Acute Respiratory Syndrome Coronavirus 2

**DOI:** 10.3201/eid2707.204660

**Published:** 2021-07

**Authors:** Mitra Gultom, Matthias Licheri, Laura Laloli, Manon Wider, Marina Strässle, Philip V’kovski, Silvio Steiner, Annika Kratzel, Tran Thi Nhu Thao, Lukas Probst, Hanspeter Stalder, Jasmine Portmann, Melle Holwerda, Nadine Ebert, Nadine Stokar-Regenscheit, Corinne Gurtner, Patrik Zanolari, Horst Posthaus, Simone Schuller, Amanda Vicente-Santos, Andres Moreira-Soto, Eugenia Corrales-Aguilar, Nicolas Ruggli, Gergely Tekes, Veronika von Messling, Bevan Sawatsky, Volker Thiel, Ronald Dijkman

**Affiliations:** Institute for Infectious Diseases, University of Bern, Bern, Switzerland (M. Gultom, M. Licheri, L. Laloli, M. Wider, M. Strässle, L. Probst, M. Holwerda, R. Dijkman);; University of Bern Department of Infectious Diseases and Pathobiology, Bern (M. Gultom, L. Laloli, M. Strässle, P. V’kovski, S. Steiner, A. Kratzel, T.T.N. Thao, H. Stalder, J. Portmann, M. Holwerda, N. Ebert, N. Stokar-Regenscheit, C. Gurtner, H. Posthaus, V. Thiel, R. Dijkman);; Institute of Virology and Immunology, Bern and Mittelhäusern, Switzerland (M. Gultom, L. Laloli, M. Strässle, P. V’kovski, S. Steiner, A. Kratzel, T.T.N. Thao, H. Stalder, J. Portmann, M. Holwerda, N. Ebert, V. Thiel, R. Dijkman);; University of Bern Graduate School for Biomedical Science, Bern (M. Gultom, L. Laloli, S. Steiner, A. Kratzel, T.T.N. Thao, L. Probst, M. Holwerda);; Institute of Veterinary Bacteriology, University of Bern, Bern (M. Strässle);; Institute of Animal Pathology, University of Bern, Bern (N. Stokar-Regenscheit, C. Gurtner, H. Posthaus);; Clinic for Ruminants, Vetsuisse Faculty, University of Bern, Bern (P. Zanolari);; University of Bern Department of Clinical Veterinary Medicine, Bern (S. Schuller);; Virology-Research Center for tropical diseases (CIET), University of Costa Rica, Montes de Oca, Costa Rica (A. Vicente-Santos, A. Moreira-Soto, E. Corrales-Aguilar);; Institute of Virology, Charité-Universitätsmedizin Berlin, Corporate Member of Freie Universität Berlin, Humboldt-Universität zu Berlin, and Berlin Institute of Health, Berlin, Germany (A. Moreira-Soto);; Institute of Virology, Justus Liebig University Giessen, Giessen, Germany (G. Tekes);; Federal Institute for Vaccines and Biomedicines, Langen,; Germany (V. von Messling, B. Sawatsky)

**Keywords:** animal experimentation ethics, coronavirus disease, disease reservoirs, epidemics, epithelial cells, outbreaks, respiratory infections, SARS-CoV-2, severe acute respiratory syndrome 2, viruses, whole genome sequencing, zoonoses

## Abstract

Severe acute respiratory syndrome coronavirus 2 (SARS-CoV-2) has spread globally, and the number of worldwide cases continues to rise. The zoonotic origins of SARS-CoV-2 and its intermediate and potential spillback host reservoirs, besides humans, remain largely unknown. Because of ethical and experimental constraints and more important, to reduce and refine animal experimentation, we used our repository of well-differentiated airway epithelial cell (AEC) cultures from various domesticated and wildlife animal species to assess their susceptibility to SARS-CoV-2. We observed that SARS-CoV-2 replicated efficiently only in monkey and cat AEC culture models. Whole-genome sequencing of progeny viruses revealed no obvious signs of nucleotide transitions required for SARS-CoV-2 to productively infect monkey and cat AEC cultures. Our findings, together with previous reports of human-to-animal spillover events, warrant close surveillance to determine the potential role of cats, monkeys, and closely related species as spillback reservoirs for SARS-CoV-2.

During the past 2 decades we have observed zoonotic outbreaks of severe acute respiratory syndrome coronavirus (SARS-CoV) in 2003 and Middle East respiratory syndrome coronavirus (MERS-CoV) in 2012 (*1*,*2*). These outbreaks have been followed by the current pandemic caused by the 2019 zoonotic emergence of severe acute respiratory syndrome coronavirus 2 (SARS-CoV-2), the etiologic agent of coronavirus disease (COVID-19) (*3*,*4*). Humans are currently seen as the main hosts, but the zoonotic origins and intermediate and potential spillback host reservoirs of SARS-CoV-2 are not yet well defined. Several reports indicate that SARS-CoV-2 spillover events from human to other animal species can occur (*5*–*7*). These zoonotic events are likely driven by close human–animal interactions and the conservation of crucial receptor binding motif (RBM) residues in the angiotensin-converting enzyme 2 (ACE2) orthologs, potentially facilitating SARS-CoV-2 entry (*8*,*9*). This knowledge gap highlights the need to assess the potential host spectrum for SARS-CoV-2 to support current pandemic mitigation strategies.

Besides their use in determine the host spectrum, animal models will be needed for viral pathogenesis studies, as well as for testing novel antiviral drugs, immunotherapies, and vaccines against SARS-CoV-2. Typically, in such studies a large variety of animal species are tested for susceptibility (*10*–*12*). However, such experiments have several drawbacks, including the availability of diverse animal models and the need for dedicated personnel, housing facilities, and most important, ethics approval. Some of these factors are especially limiting when applied to wildlife and livestock animals, such as pigs, cattle, and other ruminants; when working with companion animals and nonhuman primates, there are additional socioemotional and ethical considerations.

In this study, we evaluated the susceptibility of several mammal species to SARS-CoV-2 by recapitulating the initial stages of infection in a controlled in vitro model, in compliance with the reduction, refinement, and replacement principles in animal experimentation, while at the same time circumventing traditional in vivo experimental constraints. We used a unique well-differentiated airway epithelial cell (AEC) culture repository from the primary tracheobronchial airway tissue of 12 mammal species comprising companion animals, animal model candidates, livestock, and wild animals to assess their susceptibility to SARS-CoV-2 infection. To control for the quality of the AEC, we used influenza viruses that have known broad host tropism (*13*–*15*).

## Materials and Methods

### Conventional Cell Culture

We cultured Vero E6 cells in Dulbecco Modified Eagle medium supplemented with 10% volume/volume percent (vol/vol) heat-inactivated fetal bovine serum, 1 mmol/L sodium pyruvate, 1x GlutaMAX, 100 μg/mL streptomycin, 100 IU/mL penicillin, 1% vol/vol nonessential amino acids, and 15 mmol/L HEPES buffering agent (GIBCO; https://www.thermofisher.com). We maintained cells at 37°C in a humidified incubator with 5% CO_2_.

### Establishment of Animal AEC Culture Repository

We isolated tracheobronchial epithelial cells from 12 different animal species from postmortem tracheobronchial tissue that was obtained from slaughterhouses, veterinary hospitals, or domestic or international research institutes that euthanize their animals for diagnostic purposes or as part of their licensed experimental work in accordance with local regulations and ethics guidelines. We isolated and cultured the cells as described elsewhere (*16*). To establish well-differentiated AEC cultures from diverse mammal species, we introduced several modifications to the composition of the air-liquid interface (ALI) medium (Table). We maintained all animal ALI cultures at 37°C in a humidified incubator with 5% CO_2_. While the differentiated ALI cultures were developing over 3–4 wk, we changed media every 2–3 d.

**Table:** Optimized epidermal growth factor, retinoic acid, hydrocortisone, and DAPT concentration in the air-liquid interface medium for differentiation of the animal airway epithelial cell cultures*

Animal species	No. donors	Source	End concentration additives in ALI medium			
			EGF	RA	HC	DAPT
Monkey (*Rhesus macaque*)	2	Paul-Ehrlich-Institut, Langen, Germany	5 ng/mL	50 nM	0.48 μg/mL	NS
Ferret (*Mustela putorius furo*)	2	Paul-Ehrlich-Institut, Langen, Germany	12.5 ng/mL	100 nM	0.48 μg/mL	2.5 μM
Cat (*Felis catus*)	2	Justus-Liebig-University, Giessen, Germany	25 ng/mL	50 nM	0.072 μg/mL	NS
Dog (*Canis lupus familiaris*)	1	Institute of Animal Pathology, Bern, Switzerland	25 ng/mL	50 nM	0.072 μg/mL	NS
Rabbit (*Oryctolagus cuniculu*s)	1	Slaughterhouse, Bern, Switzerland	25 ng/mL	50 nM	0.48 μg/mL	NS
Pig (*Sus scrofa domesticus*)	2	Institute of Virology and Immunology, Mittelhäusern, Switzerland	25 ng/mL	70 nM	0.072 μg/mL	NS
Cattle (*Bos taurus*)	1	Institute of Animal Pathology, University of Bern, Switzerland	25 ng/mL	50 nM	0.48 μg/mL	2.5 μM
Goat (*Capra aegagrus hircus*)	2	Slaughterhouse, Bern, Switzerland	12.5 ng/mL	50 nM	0.48 μg/mL	2.5 μM
Bactrian camel (*Camelus bactrianus*)	1	Institute of Animal Pathology, University of Bern, Switzerland	5 ng/mL	50 nM	0.072 μg/mL	NS
Llama (*Llama glama*)	2	Institute of Animal Pathology, University of Bern, Switzerland	5 ng/mL	50 nM	0.072 μg/mL	NS
Bat (*Sturnira lilium*)	1	Costa Rica, (CIET-315–2013; permit 1841/14)	5 ng/mL	50 nM	0.48 μg/mL	NS
Bat (*Carollia perspicillata*)	1	Costa Rica (CIET-315–2013; permit 1841/14)	5 ng/mL	50 nM	0.48 μg/mL	NS

*ALI, air-liquid interface; DAPT, N-[N-(3,5-difluorophenacetyl-l-alanyl)]-S-phenylglycine t-butyl ester (a γ-secretase inhibitor); EGF, epidermal growth factor; HC, hydrocortisone; NS, not added as supplement; RA, retinoic acid.

### Virus Propagation

We propagated SARS-CoV-2 (SARS-CoV-2/München-1.1/2020/929) virus stock in Vero E6 cells for 48 h then cleared virus-containing supernatant from cell debris by centrifuging for 5 min at 500 × g before aliquoting and storing it at –80°C. We determined viral titer by plaque forming unit (PFU) assay on Vero E6 cells as described elsewhere (*17*). We prepared working stocks of influenza A virus (IAV) A/Hamburg/4/2009 strain in the pHW2000 reverse genetic backbone by propagating the rescued virus in MDCK-II cells for 72 h in the infection medium, which was composed of Eagle Minimum Essential Medium, supplemented with 0.5% of bovine serum albumin, 100 μg/mL streptomycin and 100 IU/mL penicillin solution, 1 μg/mL trypsin acetylated from bovine pancreas (Sigma-Aldrich, https://www.sigmaaldrich.com), and 15 mmol/L HEPES buffer. We determined viral titer by 50% tissue culture infective dose (TCID_50_) assay on MDCK-II cells as described elsewhere (*18*,*19*). We propagated influenza D virus (IDV, D/bovine/Oklahoma/660/2013 strain) stocks in the human rectal tumor cell line HRT-18G (ATCC [American Type Culture Collection] CRL11663, https://www.atcc.org) for 96 h in the infection medium, with the adjustment of using 0.25 μg/mL of trypsin. We determined viral titer by TCID_50_ assay on HRT-18G cells as described elsewhere (*20*).

### Infection of Animal AEC Cultures

We infected well-differentiated AEC cultures from 12 different species with 30.000 PFU of SARS-CoV-2, or 10.000 TCID_50_ of either IAV or IDV, as described elsewhere (*16*). We monitored progeny virus release at 24-h intervals for 96 h, through the application of 100 μL of HBSS onto the apical surface and incubated 10 min before collection. We diluted collected apical washes 1:1 with virus transport medium and stored them at –80°C for later analysis. After the collection of the apical washes, we exchanged the basolateral medium with fresh ALI medium. We repeated each experiment as 2 independent biologic replicates using AEC cultures established from either 1 or 2 biologic donors of each species depending on the availability of procured animal tissue (Table).

### Immunofluorescence Analysis

We fixed virus-infected animal AEC cultures with 4% vol/vol neutral-buffered formalin at 96 hours postinfection (hpi) for SARS-CoV-2 or 48 hpi for IAV- or IDV-infected AEC cultures and processed them as described elsewhere (*16*). To detect SARS-CoV-2, we incubated fixed animal AEC cultures with a Rockland (https://rockland-inc.com) 200-401-A50 rabbit polyclonal antibody against SARS-CoV nucleocapsid protein, which has previously been shown to cross-react with SARS-CoV-2 (*17*). We used an Abcam (https://www.abcam.com) ab128193 mouse antibody against IAV clone C43 nucleoprotein to detect IAV-infected cells and a custom-made rabbit polyclonal antibody against the nucleoprotein of influenza D/bovine/Oklahoma/660/2013 strain (GenScript, https://www.genscript.com) to detect IDV-infected cells. To visualize the distribution of ACE2 in the AEC cultures, we used Abcam ab15348 and Biorbyt (https://www.biorbyt.com) orb582208 rabbit polyclonal antibodies against ACE2. We used Alexa Fluor 488 conjugated donkey anti–rabbit or anti–mouse IgG (H + L) as secondary antibodies. We used Alexa Fluor 647-conjugated anti-β-tubulin (9F3) rabbit mAb (Thermo Fisher Scientific) to visualize cilia and Alexa Fluor 594 mouse anti-ZO1 1A12 monoclonal antibody to visualize tight junctions. We counterstained all samples using DAPI (4’,6-diamidino-2-phenylindole; Thermo Fisher Scientific) to visualize the nuclei. We performed imaging using a Thermo Fisher EVOS FL Auto 2 imaging system equipped with a plan apochromat 40×/0.95 air objective; we processed images using Fiji software packages (https://fiji.sc) (*21*) and assembled figures using the FigureJ plugin (https://github.com/mutterer/figurej) (*22*). We adjusted brightness and contrast of images to be identical to their corresponding controls.

### Quantitative Real-Time Reverse Transcription PCR

We extracted viral RNA from 100 μL of 1:1 diluted apical wash using the NucleoMag VET (Macherey-Nagel AG, https://www.mn-net.com), according to the manufacturer’s guidelines, on a Kingfisher Flex purification system (Thermo Fisher Scientific). We amplified 2 μL of extracted RNA using TaqMan Fast Virus 1-Step Master Mix (Thermo Fisher Scientific) according to the manufacturer’s protocol. We used a forward primer, adapted from primers described elsewhere (*23*): 5′-ACAGGTACGTTAATAGTTAATAGCGTACTTCT-3′, reverse 5′- ACAATATTGCAGCAGTACGCACA-3′, and probe 5′-FAM-ATCCTTACTGCGCTTCGA-MGB-Q530-3′ (Microsynth, https://www.microsynth.ch), targeting the envelope gene of SARS-CoV-2 (GenBank accession no. MN908947.3). As a positive control, we included a serial dilution of in vitro–transcribed RNA containing regions of the RNA-dependent RNA polymerase, envelope, and N genes derived from a SARS-CoV-2 synthetic construct (GenBank accession no. MT108784) to determine the genome copy number. We performed measurements and analysis using an Applied Biosystems ABI7500 instrument and associated software package (Thermo Fisher Scientific).

### Titration of SARS-CoV-2 in the Apical Washes

To quantify SARS-CoV-2, we titrated apical washes by plaque assay on Vero E6 cells. In brief, we seeded 1 × 10^5^ cells/well in 24-well plates 1 d before titration and inoculated them with 10-fold serial dilutions of virus solutions. We removed inoculums 1 hpi and replaced them with overlay medium consisting of Dulbecco Modified Eagle Medium supplemented with 1.2% Avicel (DuPont, https://www.pharma.dupont.com), 10% heat-inactivated fetal bovine serum, 100 μg/mL streptomycin, and 100 IU/mL penicillin. We incubated cells at 37°C with 5% CO_2_ for 48 h and fixed them with 4% vol/vol neutral buffered formalin before staining with crystal violet (*24*).

### ACE2 Homology Analysis

To analyze ACE2 homology among different species, we retrieved the available ACE2 protein sequences for humans (GenBank accession no. NM_001371415.1), rhesus macaques (accession no. NM_001135696.1), cats (accession no. XM_023248796.1), ferrets (accession no. NM_001310190.1), dogs (accession no. NM_001165260.1), rabbits (accession no. XM_002719845.3), pigs (accession no. NM_001123070.1), cattle (accession no. XM_005228428.4), goats (accession no. NM_001290107.1), and Bactrian camels (accession no. XM_010968001.1). We acquired the ACE2 sequences for *Carollia perspicillata* bats from a study of SARS-CoV and SARS-CoV-2 infection among bats (*25*). We obtained the corresponding ACE2 sequences for llamas and *Sturnira lilium* bats (accession nos. MW863647 and MW863648) by reverse transcription PCR amplification of ACE2 mRNA, as described elsewhere (*26*). We performed sequence analysis and protein alignment using the ClustalW (https://www.genome.jp/tools-bin/clustalw) plugin in Geneious Prime (https://www.geneious.com) with the default settings. We selected ACE2 protein residues interacting with SARS-CoV-2 RBM based on previously described critical ACE2 residues interacting with SARS-CoV-2 receptor binding domains (*27*,*28*).

### Whole-Genome Sequencing Using Oxford Nanopore MinION

We performed sequencing on viral RNA isolated from SARS-CoV-2 stock and the 96 hpi apical washes of SARS-CoV-2–infected monkey and cat AEC cultures according to the ARTIC platform nCoV19 protocols (*29*,*30*). We used the version 2 protocol as a basis for the reverse transcription and tiled multiplex PCR reaction using the ARTIC nCoV-2019 V3 primer pool (Appendix 1), but we used the version 3 protocol for the downstream library preparation. We generated sequencing libraries using the EXP-NBD196 Native Barcoding Expansion 96 kit (Oxford Nanopore Technologies, https://nanoporetech.com) and sequenced on an Oxford Nanopore Technologies MinION R9.4.1 flow cell for 48 h, according to the manufacturer’s instructions. We used Oxford Nanopore MinION software version 20.06.4 to perform data acquisition and real-time high-accuracy basecalling. We performed demultiplexing and read filtering according to the ARTIC platform nCoV19 pipeline (https://artic.network/ncov-2019) and the experimental Medaka pipeline (https://community.artic.network/t/medaka-longshot-pipeline/107) to perform consensus calling. We aligned and further analyzed consensus sequences in Geneious 11.1.5 using SARS-CoV-2/Wuhan-Hu-1 (GenBank accession no. MN908947.3) as the reference sequence.

## Results

**Figure 1:**
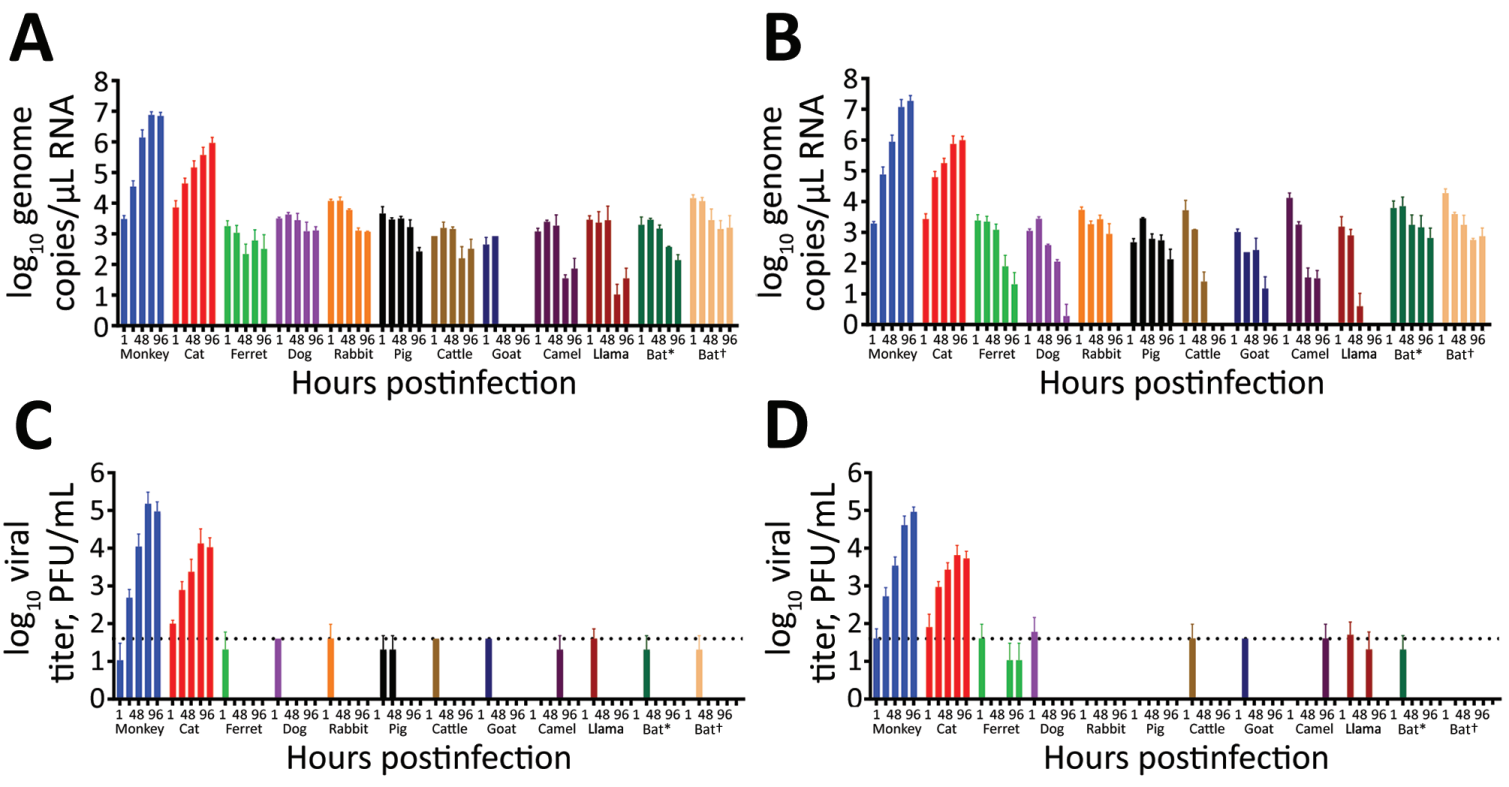
Severe acute respiratory syndrome coronavirus 2 replication kinetics in diverse mammal species. We inoculated well-differentiated animal airway epithelial cell cultures derived from the tracheobronchial epithelial cells with 30.000 PFU of severe acute respiratory syndrome coronavirus 2 at either 33°C (panels A, C) or 37°C (panels B, D). We removed inoculated virus at 1 hour postinfection and washed the apical side 3 times. We further incubated cultures for 96 h. At the indicated time postinfection, we assessed apical virus release by quantitative reverse transcription PCR targeting the E gene (panels A, B) and plaque titration assays on Vero E6 cells (panels C, D). Error bars represent the average of 2 independent biologic replicates using airway epithelial cell cultures established from 1 or 2 biologic donors. The dotted lines on panels C and D indicate the detection limit of the assay. *Sturnira lilium bat; †*Carollia perspicillata* bat.

**Figure 2:**
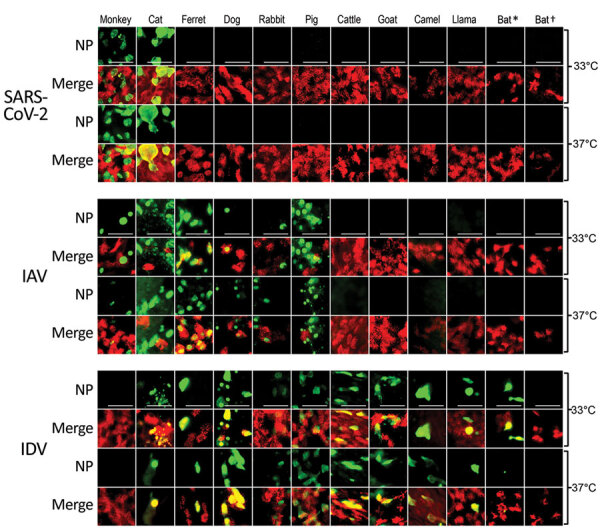
Tropisms of SARS-CoV-2, IAV, and IDV in infected airway epithelial cell cultures from diverse mammal species. We inoculated well-differentiated animal airway epithelial cell cultures with either 30.000 PFU of SARS-CoV-2 (SARS-CoV-2/München-1.1/2020/929), 10.000 50% tissue culture infective dose of IAV/Hamburg/4/2009, or IDV (D/bovine/Oklahoma/660/2013). We incubated virus-infected airway epithelial cell cultures at 33°C or 37°C and formalin-fixed them at 96 hours postinfection (for SARS-CoV-2) or 48 hours postinfection (for influenza viruses). After fixation, we stained virus-infected cultures using antibodies against either SARS-CoV-2, IAV, or IDV NP (green), and β-tubulin (cilia, red). We acquired images using an EVOS FL Auto 2 Imaging System equipped with a 40x air objective. Scale bar indicates 50 µm. *Sturnira lilium bat; †*Carollia perspicillata* bat. IAV, influenza A virus; IDV, influenza D virus; NP, nucleocapsid protein; SARS-CoV-2, severe acute respiratory syndrome coronavirus 2.

To evaluate the susceptibility of a diverse set of animal species to SARS-CoV-2 infection, we infected a total of 12 different well-differentiated mammal AEC culture models and monitored the viral replication kinetics at both 33°C and 37°C. Quantification of the viral RNA load at both temperatures showed a progressive 4-log fold increase in viral RNA load at 72 and 96 hpi in rhesus macaque and cat AEC cultures. In contrast, for the remaining animal AEC cultures we detected either a continuous or declining level of viral RNA load throughout the entire time course (Figure 1, panels A, B; Appendix 2 Figure 1, panels B, C). Because molecular assays cannot discern between infectious and noninfectious viruses, we also performed viral titration assays with the corresponding apical washes (*31*). This corroborated our previous finding that only AEC cultures from rhesus macaques and cats displayed a progressive increase in viral SARS-CoV-2 titers over time, and we detected no sustained productive virus infection above the detectable threshold beyond 24 hpi in most species (Figure 1, panels C, D; Appendix 2 Figure 1, panels D, E). The viral titers we observed in the rhesus macaque and cat AEC cultures were comparable to those we previously observed in human AEC cultures, where we also observed a 4-log fold rise in progeny-released virus in the apical side (*17*). Although ferrets have previously been shown to be susceptible to SARS-CoV-2, we observed no viral replication in AEC cultures derived from the tracheobronchial regions of ferrets. Instead, we detected only low levels of SARS-CoV-2 viral titers at 72 and 96 hpi at 37°C, in agreement with findings from in vivo studies in ferrets showing a dose-dependent and limited SARS-CoV-2 infection restricted to the upper respiratory tract (*32*–*34*). We further analyzed SARS-CoV-2 infection in the animal AEC cultures by staining for SARS-CoV-2 nucleocapsid protein on formalin-fixed AEC cultures to visualize intracellular presence of the virus. This process revealed SARS-CoV-2 antigen-positive cells in rhesus macaque and cat AEC cultures at 96 hpi, but no SARS-CoV-2 antigen-positive cells were observed in the other animal AEC cultures, including those of ferrets (Figure 2; Appendix 2 Figure 1, panel A). This further confirmed that only monkey and cat AEC support efficient replication of SARS-CoV-2 among the animals we studied.

Because productive progeny virus production was only observed in the well-differentiated tracheobronchial epithelial cell cultures from rhesus macaques and cats, we wondered whether this was because of incompatibility with the cellular receptor used by SARS-CoV-2 for cellular entry (*27*,*35*). To assess whether this corresponds to the amino acid sequence conservation of RBM in ACE2, we performed in silico analysis on the ACE2 protein sequences of the species included in this study (*27*,*28*). This process revealed that the amino acid identity of the ACE2 RBM regions interacting with the receptor-binding domain of SARS-CoV-2 in humans were more similar to those in rhesus macaques and cats than in other species (Appendix 2 Figure 2, panel A).

Apart from receptor compatibility as a limiting factor for virus infection, it has been demonstrated previously that partially differentiated AEC cultures are poorly permissive to respiratory virus infection (*36*). To investigate whether the lack of replication in ferret cells, for example, was not caused by poor differentiation of our cell cultures, we validated the AEC cultures by infecting culture samples with the 2009 pandemic IAV A/Hamburg/4/2009 and ruminant-associated IAV D/bovine/Oklahoma/660/2013 strains. Both viruses are members of *Orthomyxoviridae* and are known to have a broad host spectrum, including ferrets (*13*–*15*,*37*). We inoculated the AEC cultures from 12 different species (rhesus macaque, cat, ferret, dog, rabbit, pig, cattle, goat, llama, camel, and 2 neotropical bats) with 10.000 TCID_50_ of either IAV or IDV and incubated them at either 33°C or 37°C. At 48 hpi, we fixed the AEC cultures and processed them by immunofluorescence assays. This analysis showed that, in contrast with SARS-CoV-2 testing results, IAV antigen-positive cells could be detected in AEC cultures from companion animals and from animals commonly used for testing, such as ferret, monkey, rabbit, and pigs (Figure 2; Appendix 2 Figure 1, panel A) (*38*). For IDV infections, we observed antigen-positive cells in all AEC models, except for rhesus macaques and 1 of the neotropical bat species, indicating that AEC cultures were all well-differentiated and susceptible to virus infection.

In the immunofluorescence analysis, we also incorporated an antibody against β-tubulin marker to discern ciliated and nonciliated cell populations. For both rhesus macaques and cats, SARS-CoV-2 antigen–positive cells predominantly overlapped with the nonciliated cell populations, at either incubation temperature. Using polyclonal antibodies against ACE2, we found that the cellular receptor expression in rhesus macaques and cats predominantly overlapped with SARS-CoV-2 cell tropism, similar to ACE2 distribution in human AEC cultures (Appendix 2 Figure 2, panel B) (*17*). Unfortunately, because of limited availability of well-differentiated AEC cultures, we could not assess the ACE2 expression in goat, cattle, and rabbit AEC cultures. Nevertheless, for most species, including ferrets, that did not support efficient replication of SARS-CoV-2, we observed that ACE2 was expressed on the cell surface (Appendix 2 Figure 2, panel B). This finding suggests that ACE2 expression alone does not per se confer permissiveness to SARS-CoV-2.

**Figure 3:**
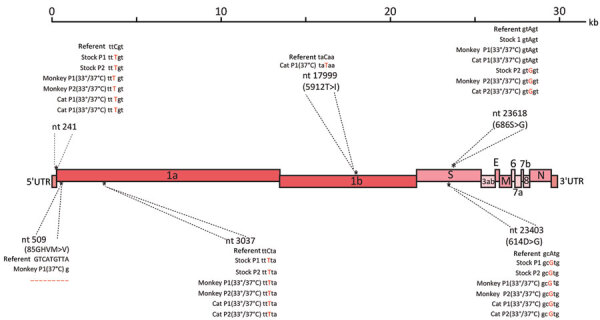
Whole-genome sequencing analysis using Nanopore sequencing technology (Oxford Nanopore Technologies, https://nanoporetech.com). A graphical representation of variants found in the severe acute respiratory syndrome coronavirus 2 (SARS-CoV-2) stock P1 and P2, as well as the apical washes from SARS-CoV-2–infected monkey and cat airway epithelial cell cultures with either P1 or P2 stock 96 hpi at 33°C or 37°C. SARS-CoV-2/Wuhan-Hu-1 (GenBank accession no. MN908947.3) was used as the reference sequence. P, passage; UTR, untranslated region.

It has previously been shown that SARS-CoV-2 can undergo rapid genetic changes in vitro (*39*). Because we observed efficient replication in rhesus macaque and cat AEC cultures, we assessed whether any mutations suggestive of viral adaptation had occurred. We performed whole-genome sequencing (Oxford Nanopore Technologies) on the viral inoculum used, as well as on the progeny viruses collected from the rhesus macaque and cat AEC cultures incubated at 33°C or 37°C after 1 passage, at 96 hpi. This inoculum was from either passage 1 or passage 2 virus stocks from the SARS-CoV-2/München-1.1/2020/929 isolate we had received. In the viral sequences in the 96 hpi samples from virus-infected rhesus macaque and cat AEC cultures, we observed no obvious signs of nucleotide transitions that led to nonsynonymous mutations compared to the respective inoculums (Figure 3), regardless of temperature and animal species. This finding highlights that the currently circulating SARS-CoV-2 D614G variant can productively infect rhesus macaque and cat AEC.

## Discussion

Our study used an in vitro AEC culture repository composed of various domestic and wildlife animal species to assess the spectrum of potential intermediate and spillback host reservoirs for SARS-CoV-2. Inoculation of AEC cultures from rhesus macaque, cat, ferret, dog, rabbit, pig, cattle, goat, llama, camel, and 2 neotropical bat species with SARS-CoV-2 revealed that tracheobronchial cells only from rhesus macaque monkeys and cats supported efficient replication of SARS-CoV-2. Whole-genome sequencing indicated that the currently circulating SARS-CoV-2 D614G variant can efficiently infect rhesus macaque and cat AEC. Our data highlight that these 2 animals are potential models for evaluating therapeutic mitigation strategies for circulating viral variants. Our findings, in conjunction with information from previously documented spillover events, indicate that close surveillance of these animals and closely related species, whether in the wild, captivity, or households, is warranted.

To date, there have been several reports published evaluating the suitability of animal models, including cats, rhesus macaques, dogs, pigs, rabbit and ferrets, for testing SARS-CoV-2 infection (*32*,*33*,*40*–*43*). We observed that SARS-CoV-2 did not efficiently replicate in tracheobronchial AEC derived from rabbits and ferrets, although ferrets are used as an animal model for SARS-CoV-2. This finding may be because viral infections in rabbits and ferrets are mainly restricted to the nasal conchae, are dose-dependent and, in addition, the origin of the cells used as input for the AEC cultures may not recapitulate the cells of the nasal mucosa (*34*,*40*,*42*,*43*). Differences exist in cellular composition and host determinant expression levels along proximal and distal regions of the respiratory tract (*44*). In addition, SARS-CoV-2 might use a different cellular receptor in ferrets, although ACE2 could be detected on the cell surface (Appendix 2 Figure 2, panel B) (*45*). Therefore, it would be of interest to complement our current repository with AEC cultures from different anatomic regions of animals such as rabbits and ferrets and to evaluate whether ACE2 is the cellular receptor employed by SARS-CoV-2 in these various animal species.

It has been proposed that SARS-CoV-2 spillover into humans, as with SARS-CoV, originally occurred from bats, either directly or through an intermediate reservoir (*3*,*46*). With >1,400 bat species comprising >20% of all mammal species, we restricted our experiments with SARS-CoV-2 to our established AEC cultures from the 2 neotropical bat species *C. perspicillata* and *S. lilium* (M. Gultom et al., unpub. data). We showed that these 2 neotropical bats express ACE2 but are not susceptible to SARS-CoV-2, suggesting that they are not likely reservoir hosts for SARS-CoV-2 despite the detection of other coronaviruses and presumptive ACE2 receptor usage by SARS-CoV-2 in closely related bat species (*25*,*47*). In fact, a 2020 study described susceptibility to SARS-CoV-2 infection in fruit bats (*Rousettus aegyptiacus*) (*33*). Future research should therefore include AEC cultures from this bat species, as well as from horseshoe bat species (genus *Rhinolophus*), which have previously been characterized as reservoir hosts for viruses with a close genetic relationship to the coronavirus associated with the 2003 SARS outbreak (*33*,*46*). In summary, our results highlight that in vitro well-differentiated animal AEC culture models can be used as an alternative to traditional animal experimentation models to evaluate and provide insight into the host spectrum of SARS-CoV-2.

Appendix 1 Primer pool for study of use of animal epithelial cells to determine animal species’ susceptibility to severe acute respiratory syndrome coronavirus 2.

Appendix 2 Additional information about the use of animal epithelial cells to determine animal species’ susceptibility to severe acute respiratory syndrome coronavirus 2.
