# Supplementary material for: Susceptibility of Well-Differentiated Airway Epithelial Cell Cultures from Domestic and Wild Animals to Severe Acute Respiratory Syndrome Coronavirus 2
Source: Emerg Infect Dis. 2021 Jul;27(7):1811–20. doi: 10.3201/eid2707.204660 (PMC8237902; doi:10.3201/eid2707.204660)
Supplement: Appendix 2 — Additional information about the use of animal epithelial cells to determine animal species’ susceptibility to severe acute respiratory syndrome coronavirus 2. [file 20-4660-Techapp-s2.pdf]

Article DOI: https://doi.org/10.3201/eid2707.204660

Susceptibility of Well-Differentiated Airway Epithelial Cell Cultures from Domestic and Wild Animals to Severe Acute Respiratory Syndrome Coronavirus 2

Appendix 2

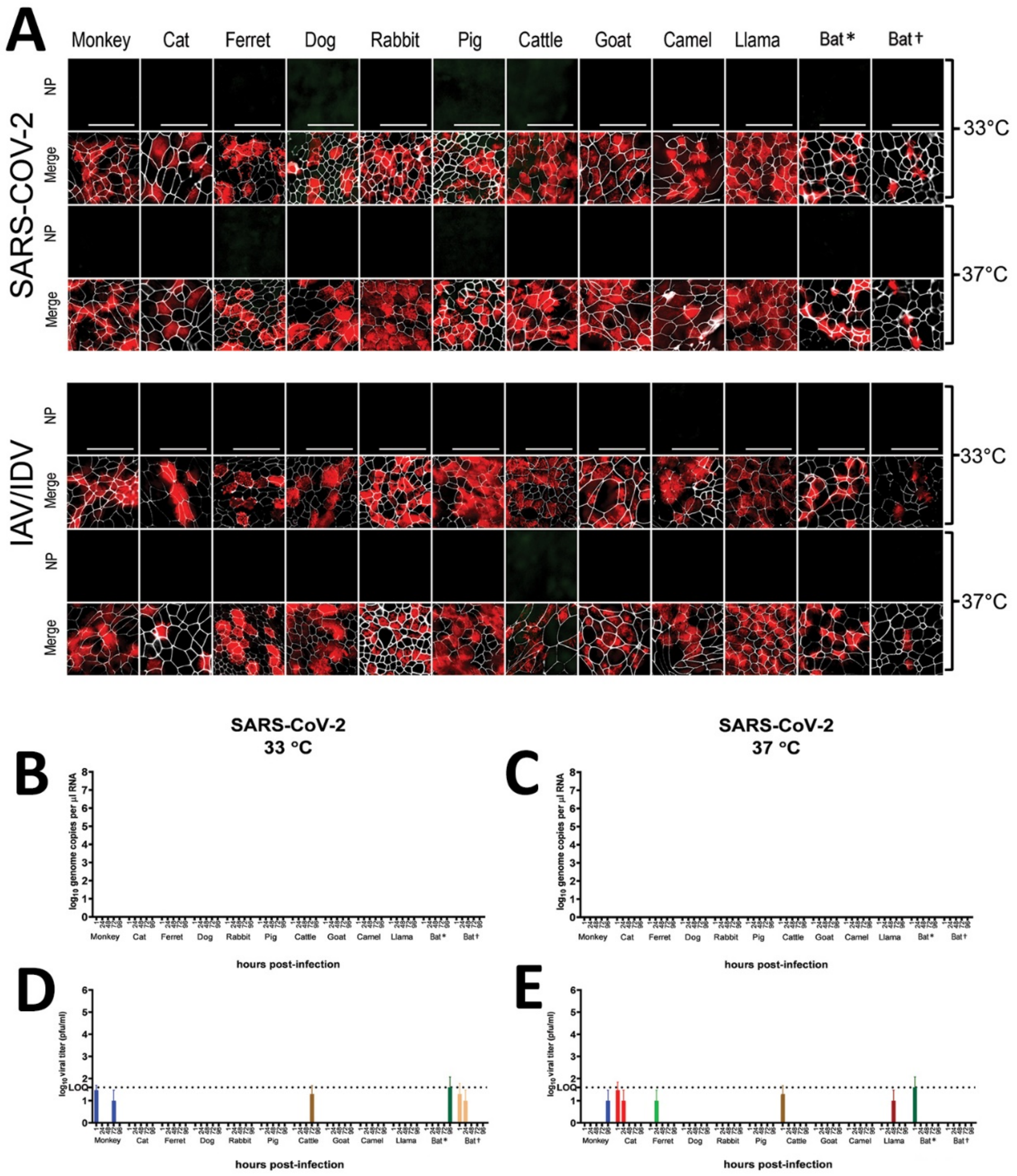

Appendix 2 Figure 1. Mock-treated cells from animal airway epithelial cell (AEC) cultures infected with severe acute respiratory syndrome coronavirus 2 (SARS-CoV-2), influenza A virus (IAV), and influenza D virus (IDV). Mock-treated AEC cultures were incubated at 33°C or 37°C in parallel with

virus-infected cells for 96 hpi (for SARS-CoV-2) or 48 hpi (for IAV and IDV). Afterwards, cells were fixed and stained using antibodies against either SARS-CoV, IAV, or IDV nucleocapsid proteins (green), β-tubulin in cilia (red), and tight-junctions (white) (panel A). Images were acquired using an EVOS FL Auto 2 imaging system (Thermo Fisher Scientific, https://www.thermofisher.com) equipped with a 40x air objective. Scale bar = 50 μm. In parallel with the SARS-CoV-2–infected cells, apical washes from the mock-treated cells were collected every 24 hours and analyzed by qualitative reverse transcription PCR (panels B,C) and plaque titration assays on Vero E6 cells (panels D,E). Error bars represent the average of 2 independent biological replicates using AEC cultures established from 1 or 2 biological donors. The dotted lines on panels D and E indicate the detection limit of the assay. IAD, influenza A virus; IDV, influenza D virus, LOA, limit of assay; NP, nucleoprotein; SARS-CoV-2, severe acute respiratory syndrome coronavirus 2; *, *Sturnira lilium*; †, *Carollia perspicillata*

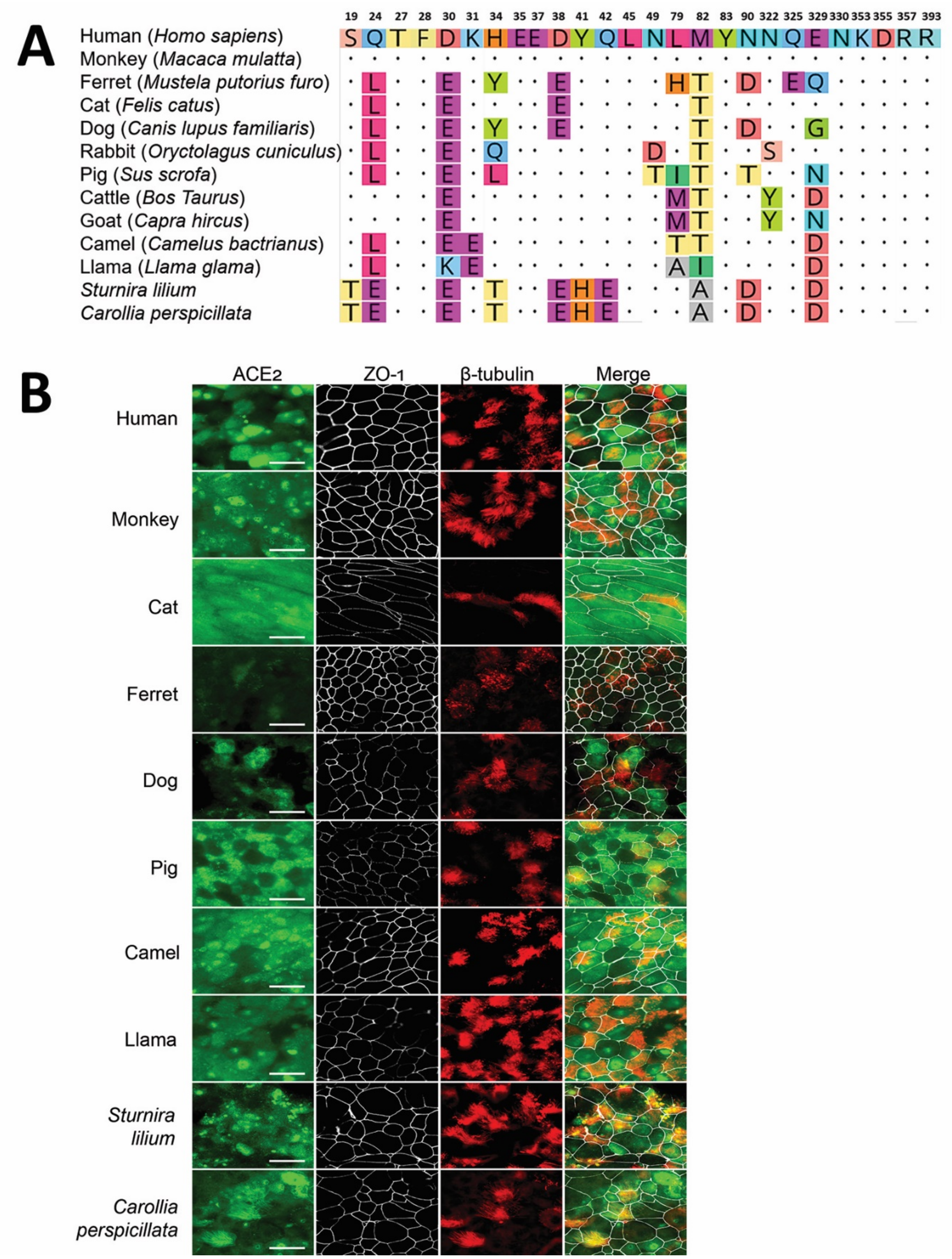

Appendix 2 Figure 2. Angiotensin-converting enzyme 2 (ACE2) analysis among different animal species. A) Protein sequence alignment of ACE2 from diverse animals in the residues interacting with severe acute respiratory syndrome coronavirus 2. The alignment was constructed using ClustalW program (https://www.genome.jp/tools-bin/clustalw). B) To visualize the ACE2 distribution in the animal airway epithelial cell cultures, formalin-fixed cells were stained with antibodies against ACE2 (green), β-tubulin (cilia, red), and ZO-1 (tight junctions, white). Image acquisition was performed using an EVOS FL Auto 2 Imaging System equipped with a 40x air objective. Scale bar is 20 μm.
